# Depoliticisation, Resilience and the Herceptin Post-Code Lottery Crisis: Holding Back the Tide

**DOI:** 10.1111/1467-856X.12060

**Published:** 2015-11

**Authors:** Matthew Wood

**Keywords:** depoliticisation, politicisation, resilience, health, Herceptin

## Abstract

Research Highlights and Abstract

This article:

- Covers new empirical terrain in the study of depoliticisation, with an in-depth case study of health technology regulation;
- Analyses depoliticisation from a novel analytical perspective, examining how depoliticised institutions are resilient to external pressure for politicisation;
- Posits a distinctive framework for analysing resilience, drawing on cognate literatures on policy networks and agencification;
- Raises interesting and distinctive questions about the nature of depoliticisation in advanced liberal democracies, arguing it is more contested than commonly acknowledged.

Depoliticisation as a concept offers distinctive insights into how governments attempt to relieve political pressures in liberal democracies. Analysis has examined the effects of depoliticisation tactics on the public, but not how those tactics are sustained during moments of political tension. Drawing on policy networks and agencification literatures, this article examines how these tactics are resilient against pressure for politicisation. Using an in-depth case study of the controversial appraisal of cancer drug Herceptin in 2005/6 by the National Institute for Health and Clinical Excellence (NICE), the article examines how ‘resilient’ NICE was to external politicisation. It is argued that NICE was resilient because it was effectively ‘insulated’ by formal procedures and informal norms of deference to scientific expertise. This mechanism is termed ‘institutional double glazing’. The conclusion suggests developments to the conceptual and methodological framework of depoliticisation, and highlights theoretical insights into the nature of ‘anti-politics’ in contemporary democracies.

The concept of depoliticisation has recently become popular in political science (Wood and Flinders 2014), describing attempts by politicians to ‘place at one remove the political character of decision making’, retaining arm's length control of policy whilst simultaneously foregoing responsibility for unpopular reforms or failures (Burnham 2001, 128; Flinders and Buller, 2006). Depoliticisation is not a new phenomenon (Fawcett and Marsh 2014)—the problematique of separating ‘politics’ from ‘administration’ and questions of central control or discretion are age-old dilemmas in policy research. However, debates over the past twenty years about the alleged ‘hollowing out’ of the state have led to a heightened focus on how ministers in core executives exercise power, with many studies arguing that central state capacity is being reduced (for a critical review see Matthews 2013). Depoliticisation as a concept is interesting because it turns this common argument on its head. Situated within the broader ‘critical’ governance literature (Davies 2011) depoliticisation highlights how alleged ‘hollowing out’ through delegation to ‘arm's length’ bodies actually *enhances* politicians' capacities to institute their ideological preferences within a set of concrete rules and ‘expert’ decision making procedures (Flinders 2008). These become embedded through the process by which those decisions are made to *appear* unchallengeable or ‘non-political’ (depoliticised).

How does this process work in practice, though? Do allegedly ‘expert’ arm's length institutions remain embedded over time, and how are their decision-making processes resistant to challenge? Answering these questions involves, in part, examining how central state ministers resist pressure to intervene in ('re-politicise’) these ‘arm's length’ bodies, hence demonstrating their effective ‘institutionalisation’. Existing research has examined depoliticisation as a way of deflecting blame from politicians for policy failure (Kettell 2008) and conversely on the success of re-politicisation strategies (Kuzemko 2014). It has not, however, analysed how putatively depoliticised bodies are *resilient against* pressures for re-politicisation in external policy networks. Hence, the question this article addresses is: how do depoliticised bodies retain and maintain their depoliticised status under conditions of political stress? In addressing this question the article contributes distinctively to what Hay (2014) calls a ‘second generation’ of depoliticisation research. This diversifies into new policy areas and examines distinctive research questions, but with a continued concern for *critically* interrogating the mechanisms that often prevent us in democratic societies from seeing substantively our collective choices.

In order to achieve this aim the article posits an analytical framework for examining the tensions and stresses on depoliticised bodies, building on Hay's (2007) insights on pressures for politicisation and utilising literature on policy networks and agencification. The framework is applied to an in-depth case study of the controversial appraisal process for Herceptin, a new biomedical drug for treating early-stage HER2-positive breast cancer in 2005/6 under the British New Labour government. This case is ideal because it focuses on an ostensibly ‘depoliticised’ institutional relationship operating within a context of high public pressure. A delegated expert body—the National Institute for Health and Clinical Excellence (NICE)—was responsible for appraising Herceptin for free distribution on the National Health Service (NHS), but there was severe pressure from media and interest groups for ministerial intervention to approve the drug immediately. NICE hence provides a critical case of a ‘depoliticised’ agency in a ‘politicised’ context, and thus fertile empirical territory for uncovering the mechanisms through which the ‘depoliticised’ nature of the institution is sustained in practice. This case is also empirically distinctive in relation to existing literature on depoliticisation, which focuses almost exclusively on the economic policy field (Kettell 2008; Rogers 2009). Indeed, investigating depoliticisation in health technology regulation has arguably even greater pertinence, given the importance of long-term public trust and confidence in organisations like NICE as truly autonomous judges of clinical cost-effectiveness.

Analysing the case, it is argued that high levels of pressure from outside the state to intervene in the appraisal did not lead to intervention—politicisation—in the decision making process itself. This is because decision making was highly formalised with strict procedures of stakeholder input, and informal norms of deference to scientific expertise created what this article terms ‘institutional double glazing’ that prevented ministerial intervention. Formal and informal institutional mechanisms hence countered external politicisation to protect the depoliticised body from interference. In making this argument the article utilises qualitative and quantitative data gathered from sixteen semi-structured elite interviews with critical actors^1^ during the appraisal, analysis of government documents, media coverage, parliamentary debates and a freedom of information request.

This article is hence organised around three central ‘hooks’ or claims to distinctiveness at the micro (empirical), meso (analytical), and macro (theoretical) levels:

(1) *Micro (empirical)*: this article covers new empirical terrain in the study of depoliticisation (health technology regulation) from a novel analytical perspective (examining the resilience of depoliticised bodies).
(2) *Meso (analytical)*: this article posits a distinctive analytical framework for assessing the resilience of depoliticised bodies, developing Hay's (2007) insights into (de)politicisation processes by engaging with cognate literatures on policy networks and agencification.
(3) *Macro (theoretical)*: this article offers important insights into the nature of depoliticisation as a process, arguing it is the product of periodic contestation and resistance, rather than of top-down ideological assimilation.

In making these contributions, this article is divided into six sections. Firstly, it briefly provides a critical review of the depoliticisation literature. Secondly, it draws from the policy network and agencification literatures, and Hay's (2007) model to construct a framework for assessing resilience. The third section introduces the Herceptin case before the fourth section examines the emergence of an ‘issue network’ during the Herceptin crisis, creating pressures towards politicisation, and the fifth section analyses how NICE was resilient to this external pressure, retaining its ‘depoliticised’ status. The conclusion then argues for further methodological integration of ‘depoliticisation’ with cognate literatures, building upon Kettell's (2008) methodology for assessing depoliticisation strategies, and for further comparative analysis of the nature and form of depoliticisation in different policy areas, theorised as the institutional embedding of particular modes of policy-making in a context of periodic political struggle and contestation.

## Depoliticisation, Governance and Resilience

Academic work on depoliticisation has blossomed in recent years, particularly in relation to governance and public policy (Burnham 2001; Buller and Flinders 2005; Kettell 2008; Rogers 2009; Beveridge 2012). Depoliticisation, as defined here, is ‘the range of tools, mechanisms and institutions through which politicians can attempt to move to an indirect governing relationship and/or seek to persuade the demos that they can no longer be reasonably held responsible for a certain issue, policy field or decision’ (Flinders and Buller 2006, 295–296). Examples of depoliticisation ‘tactics and tools’ include, most prominently, the delegation of decision making to supposedly ‘non-political’ arm's length bodies (ALBs), which are widely interpreted by the public as being ‘non-political’ (Flinders 2008, ch. 7). Empirical research has focused on the success of depoliticisation (Kettell 2008; Rogers 2009; Beveridge 2012), how depoliticised policy areas are re-politicised (Kuzemko 2014) and ‘the broader relationship between depoliticising and politicising dynamics’ (Jenkins 2011, 158; Bates et al. 2014).

While existing research has begun delving into this ‘broader relationship’, it has not yet examined how the ‘indirect governing relationship’ identified by Flinders and Buller (2006) is maintained, despite external pressures for politicisation. Scholars such as Moran (2003, 139), for example, note the prevalence of ‘hyper-politicisation’, or how ‘the quango world (being) drawn inexorably into an increasingly open, partisan and juridified world’. The extent to which these ‘quangos’ are able to resist such pressures is a key factor in determining whether they are ‘successful’ in retaining their ‘depoliticised’ status over time. Such questions are important for critically analysing the barriers or obstacles to ‘seeing clearly the political choices that govern our ostensibly democratic societies’, which Hay (2014, 310) highlights as a key objective of future research. A central point of this article, then, is specifically to address the question: how do depoliticised institutions retain their depoliticised status during periods of political stress (crises)?^2^ This question is distinctive with regards to the depoliticisation literature but, as we shall see, it is situated within a much broader literature on governance and policymaking. This literature offers important tools for addressing the resilience of depoliticisation substantively.

## The Resilience of Depoliticisation: Towards an Analytical Framework

**Figure 1:**
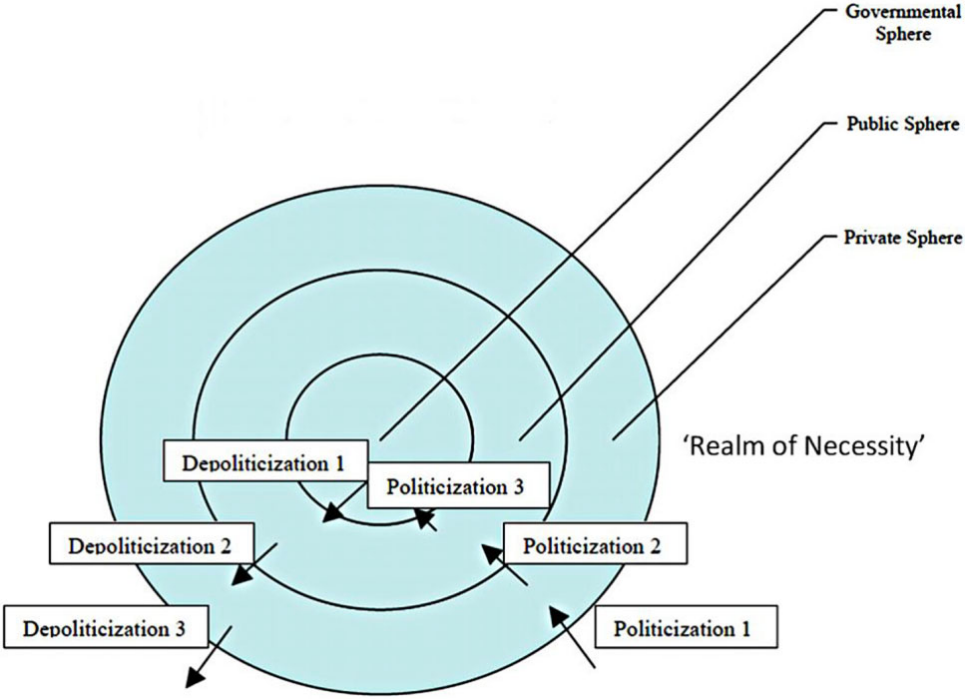
Hay's (2007) Heuristic Model of Politicisation/Depoliticisation

*Source*: Hay 2007, 80

As the previous section suggests, particular tactics of depoliticisation exist within a broader political context that contains a range of tensions and pressures for politicisation, and the aim of this article is to bring out how depoliticisation strategies are resilient against politicising pressures in a way that existing work only suggests implicitly. A useful heuristic starting point for this task is Hay's (2007) model of (de)politicisation processes, shown in Figure 1.

Hay's model posits three politicisation processes. Politicisation 1 refers to movements from the ‘realm of necessity’ where there is no capacity for human action to the ‘private’ realm as actors acknowledge humans have the capacity to govern their environment. Politicisation 2, movement from ‘private’ to ‘public’ realm occurs when an issue gains acknowledgement as an issue of collective concern through, for instance, publicity campaigns. Politicisation 3 occurs when this issue becomes of legislative interest in Parliament and the responsibility of state departments. This heuristic model suggests the ‘tactics and tools’ of depoliticisation are influenced by multiple wider processes. Moreover, it frames the systemic tensions and pressures this article seeks to analyse in an intellectually appealing and exciting single framework of (de)politicisation.

At the same time, however, Hay (2014) also cautions against fundamentally departing from established analytical approaches. Analysing how non-state actors influence the state is far from a new endeavour, as Bendix and Lipset (1957, 87) stated 50 years ago: ‘political science starts with the state and examines how it affects society, while political sociology starts with society and examines how it affects the state’. In policy analysis concepts like ‘iron triangles’, ‘policy whirlpools’, ‘venue shopping’, ‘policy streams’, ‘networks’ and ‘advocacy coalitions’ (for a review see Cairney 2012) have long proved useful for analysing how non-state actors influence government. There is also the danger of ‘conceptually stretching’ the notion of politicisation in a way that, within the confines of this article, cannot be justified. In light of these challenges this article adopts a strategy of identifying existing approaches that can be combined for analysing the resilience of depoliticisation by identifying and interrogating points of pressure or tension within the political system that Hay's model intimates. Two frameworks are included here: policy networks and agencification.

Firstly, the extensive literature on policy networks is salient in examining the *intensity or vigour* of societal pressure upon the state (Rhodes 1990; Marsh and Rhodes 1992). Policy network scholars in the ‘British’ school differentiate between ‘issue networks’ and ‘policy communities’ as forms of ‘interest intermediation’ in the transfer of policy demands towards the state, the former being ‘loose’, disorganised and open to multiple actors, and the latter being ‘tight’, professionalised and closed to outsiders (Marsh 1998). For Marsh (1998, 16), in an issue network there is greater conflict over core values and challenge of the policy status quo, whereas in policy communities ‘all participants share basic values and accept the legitimacy of (an) outcome’. Moreover, Marsh (1998, 16) highlights an increased and diversified number of actors involved in policy debates, a fluctuation of ‘access’ and contacts within the network, and less ability to ‘regulate’ or control network membership. Policy network analysis often utilises a mixed method approach, including document analysis, elite interviews, statistics of media coverage and parliamentary debates.

Despite critiques of this approach as lacking ‘explanatory power’ (Dowding 1995) policy network analysis is important because it highlights the intensity of pressure exerted upon government. Potential links with ‘politicisation’ have been noted in policy network studies (e.g. Wilkinson et al. 2010, 332). Smith (1991), for example, argues that issue networks tend to be more ‘politicised’ as they include more dissenting voices and place greater pressure upon central government. By contrast, policy communities ‘depoliticise a policy arena by excluding groups which are likely to disagree with the established policy agenda from the policy-making process’ (Smith 1991, 236). The invocation of these concepts highlights a conceptual link that might be developed between types of policy network and levels of (de)politicisation.

Secondly, literature on ‘agencification’ is also concerned with tensions and pressures, except at the intersection between arm's length bodies (ALBs) and central ministerial departments (see Gilardi and Maggetti 2011). Of particular interest here are studies which examine the extent of informal political pressure applied by central government departments on ALBs (e.g. Maggetti 2007). This work analyses the *de facto* autonomy of ALBs, or their ‘actual existing’ independence from central government. *De facto* autonomy can be defined as ‘the extent of regulators’ effective autonomy as they manage their day-to-day regulatory actions' (Gilardi and Maggetti 2011, 204). Empirical assessment can be based upon examination of semi-structured elite interviews and levels of communication between central department and delegated body, with greater communication indicating less *de facto* autonomy (Maggetti 2007). An analysis of *de facto* autonomy can be connected with (de)politicisation, given the focus on ‘measuring informal political influence’ (Flinders and Buller, 2006, 302). Where ALBs are *de facto* more autonomous, they are ‘depoliticised’, and where they are less autonomous, they become ‘politicised’.

While not the only bodies of cognate literature, these frameworks are important because they highlight dynamic tensions at key intermediary points within political systems (between public networks and central states, and between ministerial departments and ALBs). Analysing *de facto* autonomy can determine whether ‘politicisation’ has occurred when evidence shows central departmental actors either overruling ALB decisions, or increasing interactions with ALBs regarding particular decisions. If there is evidence of an issue network emerging but not of central departments intervening in ALBs, this suggests depoliticisation is ‘resilient’. Explaining this resilience contributes towards a more fine-grained analysis of the factors that prevent depoliticisation being challenged, which the remainder of this article attempts to do.

## NICE and the Herceptin Post-code Lottery Crisis

The regulation of new ‘health technologies’ (drugs, medicines, treatments, etc.) ‘in a number of ways epitomises many of the features of the late-modern British regulatory state’ (Brown and Calnan 2011, 1). In 1999, The National Institute for Health and Clinical Excellent (NICE) was established by New Labour Health Secretary Frank Dobson to take decision-making power over the funding of drugs for free prescription on the National Health Service (NHS) away from politicians. Supposedly, this would end the ‘post-code lottery’ in which only wealthy Primary Care Trusts (PCTs) could fund expensive treatments. NICE was ‘intended to ensure that the lottery system under which certain treatments and drugs are available in one part of the country but not another is changed’ (House of Commons Debate 18 January1999, 323: 592–594) and NICE was given ‘considerable control over its own organisation and rules of procedure’ to ensure this (Landwehr and Böhm 2011, 681). NICE's technology appraisal process is a key element of its autonomy, carried out in a tightly structured manner for up to two years, with little input from the Department of Health (DH) (Milewa and Barry 2005, 506). This process can be reduced to roughly six stages:

(1) The European Medicines Agency (EMEA) licenses a ‘technology’ for distribution around Europe.
(2) DH consults NICE and other groups, and then refers the technology to NICE for appraisal.
(3) NICE identifies and consults with the policy community, produces an appraisal scope/timeline and appoints a (university-based) Technology Assessment Group (TAG).
(4) The TAG writes an evaluation report of the technology's effectiveness using the methodology of cost-per Quality-Adjusted Life Years (QALYs).
(5) A Technology Appraisal Committee (TAC) comprised of practitioners from outside NICE receives evidence from the TAG and submissions from other consultees (including DH) and produces an Appraisal Consultation Document (ACD)
(6) The ACD is circulated around consultees for comments and a Final Appraisal Document is then sent to NICE's Guidance Executive for approval. PCTs have a short appeal window but must then implement guidance within 3 months (see NICE 2001; NICE 2004).

This process leaves little room for intervention from DH, with the only direct role being when it delegates the initial decision (a decision which itself is usually advised on by NICE). It can hence be argued that NICE is a good example of a ‘depoliticised’ institution with minimal central government input (Syrett 2003, 728). Yet, health technology appraisals are hardly uncontroversial, and somewhat paradoxically NICE also created ‘new layers of subjectivity and policy meddling’ (Brown and Calnan 2011, 2) as it ‘politicised questions of priority setting and rationing’ by making issues of rationing an ‘explicit’ national task as opposed to an ‘implicit’ local task (Landwehr and Böhm 2011, 680). Moran (2003, 141) argues that this is a clear example of ‘hyper-politicisation’—‘the breakdown of the old doctor-dominated systems of control, and the translation of rationing issues into increasingly open political argument’. This policy area hence presents a good opportunity for a case study—depoliticised decision making in a situation of (potentially) high pressures for politicisation—and this is particularly pertinent in the case of Herceptin itself.

Herceptin (medical name ‘trastuzumab’) is a prominent example of a ‘targeted’ biomedical drug, a new breed of drugs developed in the 1980s–90s which ‘provide the link between an individual's molecular and clinical profiles … allowing patients the opportunity to make informed and directed lifestyle decisions'—thus ‘personalising’ the medical process (Ginsburg and McCarthy 2001, 491). Herceptin targeted a particular form of breast cancer—‘HER2-positive’—which involves faster growth of tumours and a more aggressive form of the disease, with survival time as little as nine months (NICE 2002, 2). While Herceptin had previously been recommended by NICE in 2002 for late-stage HER2-positive patients, before May 2005 it had not been approved for those at earlier stages.

However, in May 2005 new findings on Herceptin's ‘early stage’ effects were announced from the Herceptin Adjuvant (HERA). International pharmaceutical distributor Roche claimed the drug had potentially radical, even curative effects, including a 50% reduction in return after early stage treatment. This amounted to ‘the first evidence that Herceptin had the potential to reduce the risk of cancer coming back at an early stage and to prolong life for women with this aggressive form of the disease’ (Roche 2005a). Hence, although doubts persisted within the medical community over negative effects upon patients with previous heart problems (Keidan 2007) Herceptin came into public view not only as a new, potentially curative treatment to a debilitating disease that could be given to patients with a high probability of success. As one interviewee stated:

Why is Herceptin interesting? Well, because it was the first of a new type of treatment that was produced so that it was a targeted therapy. You didn't have to treat all patients, you could identify which patients would do well … the response was dramatic (Interview, former Roche executive, January 2012).

As of May 2005, however, the EMEA had not licensed Herceptin for the European market, and hence NICE could not begin its appraisal process. Were Herceptin fully licensed and recommended by NICE, PCTs would be legally obliged to fund treatment to all eligible patients. Here, though, given Herceptin was unlicensed and no guidance was yet available, instances where treatment was requested were dealt with by PCT boards on a case-by-case basis. PCT boards could consider any patient requests to be provided with Herceptin for free using PCT funds, but had discretion over whether to provide funding depending upon available resources (which patients could appeal against). This led to geographical disparities in the provision of Herceptin, because of the substantial cost involved and unequal resources across PCTs, and hence a ‘post-code lottery’ emerged. Given that the ‘(elimination of the) post-code lottery of care was according to political pronouncements the raison d'ětre for the establishment of NICE’, this presented an acute predicament (Littlejohns 2001, 40). In theory, Herceptin was just one example of a systemic problem often termed ‘NICE blight’ or ‘the delay between product launch and availability of NICE guidance’ (Barham 2008, 1037). However, several interviewees noted Herceptin is widely considered ‘exceptional’ or ‘defining’ due to the extent of political interest. In this case, NICE's processes were brought into question, and ministers were under significant pressure to push through guidance and end the ‘post-code lottery’. The next section examines the empirical evidence, firstly analysing the emergence of an ‘issue network’ around Herceptin.

## Playing Out the Post-Code Lottery: Pressures for Politicisation

The Herceptin post-code lottery crisis was played out from roughly 19 May 2005 when Roche first presented evidence to the American Society of Clinical Oncologists' (ASCO) Conference, up to the final NICE appraisal released on 23 August 2006. There were three key periods: ‘crisis emergence’, ‘crisis peak’ and ‘crisis diffusion’.

### Crisis Emergence

In the first period Roche's high profile presentation of the HERA trial findings at the ASCO Conference initiated an international clamour for access to Herceptin. As one interviewee recounted:

It was a very emotional period; people were astounded that the results were better than anyone had ever anticipated. That got reported by the press in the UK as it did in every country in the world that this was the breakthrough cancer that for the first time had shown these dramatic results, and patients wanted to get hold of it (Interview, former Roche executive, January 2012).

**Figure 2:**
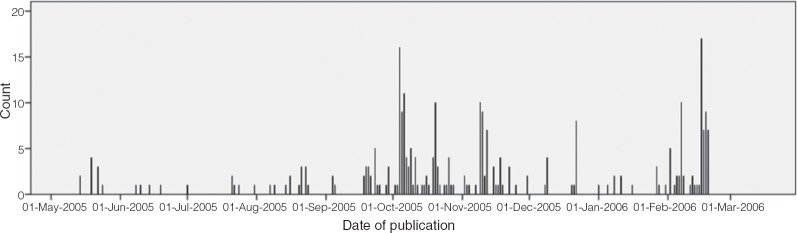
National News Articles Published on Herceptin (14 May 2005–19 February 2006)

*Source*: Graph computed by the author from data supplied by Wilson et al. 2008

The presentation created an almost euphoric media reaction. Whereas only 13 Herceptin articles had been published in national news articles since November 2003, May 2005 produced 10 articles alone, all of which had a positive slant towards Herceptin. Importantly, the first ‘case study’ of a patient, Barbara Clark, who was eligible for Herceptin but denied treatment by North Somerset PCT was also highlighted (BBC News 8 June 2005) and raised by her MP in parliament (HoC Deb 30 June 2005, 435: 1543). Already, there was pressure to conform to international trends and circumvent existing regulatory processes. The issue network at this stage, however, was not fully developed. Domestic pressure was largely localised, and, as Figure 2 (below) shows, national press coverage was relatively low.

### Crisis Peak

However, as is clear from Figure 2, from mid-September to late-November there was a much more sustained period of media pressure. National interest became an almost daily occurrence, with 24 pieces in September, 87 in October and 49 in November. One interviewee recalled the intensity:

Herceptin gave some particular difficulties for us because NICE generally wouldn't comment until we were formally working on a piece of guidance. The queries from journalists and others were coming in well before we got to that stage, putting the NICE press office and DH press office under quite a lot of pressure … It was on a daily basis, the NICE press office took around 60 calls a week from journalists just about our work, it was a really busy press office and a significant proportion of those were about Herceptin for the whole period it was going on. So Herceptin did put real pressure on the press office (Interview, former manager of NICE communications team, January 2012).

Interviewees from both DH and NICE agreed that this pre-assessment period was the most intensely ‘difficult’ period of media pressure. Here, there was an intensification of pressure group activity, particularly from long-campaigning cancer charities such as Breakthrough Breast Cancer and Cancer Bacup (Hind et al. 2011, 24) but also a more temporary pressure group, Women Fighting for Herceptin (WFH). Having featured on Radio 4's *Women's Hour* programme in August, a small group of Staffordshire patients took their campaign national, aided by public relations firm Porter Novelli (*The Guardian* 29 March 2006) and Roche's own media campaign accompanying publication of the HERA findings in October (Roche 2005b). WFH submitted a petition to Downing Street signed by 34,000 people, and were given a meeting with Health minister Rosie Winterton. The event gained national media coverage leading *The Sun* to launch an appeal urging Patricia Hewitt (Health Secretary) to ‘make Herceptin available immediately’ (*The Sun* 29 September 2005) and parliamentary motions to speed up the process (HoC Deb 21 October 2005, 437: 1270W; HoC Deb 22 November 2005, 439: 1358). As one interviewee remarked: ‘(t)here was a lot of stuff in the media, a lot of activity and these groups became very prominent and somehow struck a chord with society’ (Interview, NICE Executive Director, January 2012).

Moreover, several patients launched legal challenges against PCT decisions denying them treatment. Firstly, North Somerset PCT designated HER2-positive patient Barbara Clark an ‘exceptional case’ after she threated action in the European Court of Human Rights. The importance of this event is clear, as 3 October marked the first peak in media interest (see Figure 2), and Clark would become the most mentioned patient (Wilson et al. 2008, 128). As various patients challenged their PCTs on legal grounds following this reversal, ‘the Herceptin campaign rose to fever pitch’ (Keidan 2007). In November, North Stoke PCT and WFH member Elaine Barber threatened legal action after being denied Herceptin on grounds of cost and heaving an appeal rejected (Mayor 2005). Barber's subsequent legal threat led to the PCT agreeing to reverse its decision, on grounds of ‘exceptional circumstances’ (Mayor 2005). These mounting legal challenges, driven by expert solicitors Irwin Mitchell, provided a focal point for media attention on the Herceptin issue, as one interviewee noted: ‘The main media interest was around when the cases were being issued or the hearings or decisions. So the media tried to work the story around the cases as opposed to around the general public campaign’ (Interviewee, Solicitor, January 2012).

### Crisis Diffusion

In February, media coverage peaked as Swindon PCT patient Ann Marie Rogers became the first to take her appeal to the High Court. When Rogers' case was rejected (Dyer 2006), a storm of negative media coverage followed (e.g. BBC News 15 February 2006, see Figure 2). The percentage of articles criticising the ‘post-code lottery’ reached its highest since May 2005 (77.9%), and there was only one ‘positive’ article that month towards the ability of patients to access Herceptin, out of 68. A Panorama programme entitled ‘Wanting the Wonder drug’ also created negativity, emphasising the personal experiences of participants in the WFH campaign (BBC Panorama 7 February 2006).

By now, however, the appraisal process was already underway, as NICE had appointed the Evidence Review Group (ERG) for assessing Herceptin, based around a new ‘Single Technology Appraisal’ (see below) that started the appraisal process in line with, rather than after the licensing decision. On 7 February NICE received evidence on Herceptin from Roche and on 17 February Roche applied to the EMEA for a European license. Significant media coverage and Parliamentary debate on the topic continued through April (Hind et al. 2011, 42), as the Court of Appeal's ‘landmark judgement’ on 12 April overturned the High Court's ruling on Ann Marie Rogers (*The Independent* 13 April 2006). However, because the regulatory process was already underway, with licensing expected by late-May and a NICE decision shortly afterwards, the issue's salience decreased.

This diffusion of pressure continued through to the later stages of the assessment process, as Hind et al. (2011, 42) and Abelson and Collins (2009, e120) show. Although Newbury and Community PCT appealed against NICE's draft guidance published on 9 July, this was swiftly rejected by a NICE review panel (NICE 2006a) and final guidance was issued on 23 August recommending Herceptin for treatment ‘for women with early-stage HER2-positive breast cancer following surgery, chemotherapy (neoadjuvant or adjuvant) and radiotherapy (if applicable)’ (NICE 2006b, 4).

The above analysis suggests that a distinctive ‘issue network’ emerged around Herceptin during its appraisal. It is clear that there was a substantial increase in the salience of Herceptin as an issue in the wider public sphere, and the participation of various legal, medical, political, journalistic and marketing groups in pressurising government over the issue. This shows evidence of the emergence of an ‘issue network’ based on a more critical and diverse environment of actors. Moreover there were also more contests over legitimacy typical of an issue network, in the sense that debates within the network became critical of existing governance arrangements. This amounts to a significant amount of pressure from the network on ministers to intervene in NICE's appraisal and ‘fast track’ Herceptin. The remainder of this article shows, however, that NICE was arguably very resilient to this pressure.

## Maintaining Depoliticisation

Publicly declared actions of ministers developed roughly in line with political pressures. In the ‘emergence’ crisis period the primary action was to refer Herceptin to NICE. On 21 July Herceptin was referred outside the normal referral ‘waves’ of health technologies (NICE 2005b). At this early stage, however, Herceptin was not referred to as an ‘exceptional’ case, and DH referred Herceptin alongside Velcade, another high profile drug used to treat Multiple Myeloma. Under-Secretary for Health Liam Byrne was thus able to respond to controversy surrounding Barbara Clark by stating that existing processes would remain:

It is perhaps inappropriate for NICE to determine the clinical effectiveness and cost-effectiveness of a drug while its safety and efficacy are still under consideration … we cannot override the drug licensing process (HoC Deb 30 June 2005, 435: 1548–1549).

At this stage DH was seen as performing its primary function, namely, referring drugs to NICE, and nothing more. More crucial is the ‘peak’ stage, in which ministerial activity increased significantly. The most important action here was Patricia Hewitt's comment on 5 October, at the first peak of media pressure:

Herceptin has the potential to save many women's lives and I want to see it in widespread use on the NHS … I want the licence for Herceptin to be granted as quickly as possible … and to be available within weeks of the licence being given. I share the huge frustration of many women about the delays in getting Herceptin licensed. I am determined to take action, and this represents a major step forward in our fight against cancer (DH 2005).

This announcement led to further ‘interventions’. On 25 October the Health Secretary made similar remarks to the Breakthrough Breast Cancer Fly-in, this time compelling PCTs to fund Herceptin outside of NICE recommendations:

I have shared the huge frustration of many women about the delays in accessing cancer drugs … it has the potential to save as many as 1000 lives a year … I want to make it clear that PCTs should not refuse to fund Herceptin solely on the grounds of cost … I have asked (NICE) to start on a fast-track appraisal (Hewitt 2005).

Prime Minister Tony Blair even suggested PCTs should ‘go ahead and allow people to use (Herceptin)' (BBC News 3 November 2005).

Given the ‘unusual and exceptional’ amount of ministerial activity, as one interviewee commented, it could be argued that ministers were essentially predetermining the assessment process (Mayor 2005). Such arguments only gain greater salience when we consider that shortly after Blair and Hewitt's statements NICE introduced a ‘Single Technology Appraisal’ (STA) process as a way to ‘enable single new drugs, and existing drugs with new indications to be rapidly assessed’ (NICE 2005a). Herceptin was the first drug to go through this quicker process which ran alongside, rather than beginning after, the licensing decision by the EMEA, hence aiming to close the gap in which a ‘post-code lottery’ could exist (for a full description see NICE 2006c). That the introduction of the STA followed shortly after ministerial statements about a desire to ‘speed up’ the appraisal process certainly intimates some form of ‘political’ influence on NICE's decision making, even suggesting ministers leaning on NICE officials to achieve a positive appraisal. However, close analysis of documents provided via a freedom of information request for all correspondence between NICE and DH regarding Herceptin from 1 May 2005 to 1 September 2006 suggests this is not the case. This data is presented in Table 1.

**Table 1:** Email Communications between DH and NICE Referring to Herceptin (1 May 2005–1 September 2006)

		Subject			
		
Month	No. of Email Communications	Referral	Press handling	Consultation	Other
May 2005	1	1	—	—	—
June 2005	1	1	—	—	—
July 2005	3	2	1	—	—
August 2005	1	1	—	—	—
September 2005	0	—	—	—	—
October 2005	3	—	3	—	—
November 2005	5	—	5	—	—
December 2005	2	—	—	2	—
January 2006	0	—	—	—	—
February 2006	0	—	—	—	—
March 2006	2	—	—	—	2
April 2006	1	—	—	1	—
May 2006	1	—	—	1	—
June 2006	2	—	1	1	—
July 2006	1	—	—	1	—
August 2006	2	—	—	2	—

Table 1 shows the number of emails exchanged between NICE and DH explicitly mentioning Herceptin is very small. Even during the most intense periods of pressure in October and November only a total of 8 emails explicitly mentioning Herceptin were sent. The email subjects provided in columns 3–7 from the left also suggest that the nature of this email communication was largely formalistic in nature. Emails from May–August focused largely upon the formal referral of Herceptin, specifically NICE advising DH within a Joint Planning Group (JPG). There is a significant exchange around the end of October regarding the coordination of the STA launch (would it be a joint DH and NICE statement or separate ones?) and stock responses for media questions on Herceptin. The clear theme, however, is not the decision to implement STA itself, but *coordination*—who will say what, how will the image of NICE independence be maintained and how will Herceptin be downplayed as a driver of the new process. This press handling subsides as the STA appraisal for Herceptin begins, and the majority of communication from December 2005–August 2006 relates to DH's formal role as a consultee in the STA.

The data presented above suggests that contact (in email) was limited mainly to formal communication in terms of DH's referral and consultation roles. This finding is supported by a NICE response to a House of Commons Select Committee report on its activities:

There has never been any direct or indirect attempts by ministers to influence our guidance once topics have been referred for consideration. Sometimes we are asked to consider issues that generate significant public interest and comment; and ministers may give interim advice to the NHS on how to manage such issues while we are developing formal recommendations. While our independent advisory committees are aware of interim advice from ministers, this advice does not influence the formal recommendations that they develop (House of Commons Health Committee 2008, 1).

This argument is reinforced by the evidence of interviewees from both DH and NICE. As one interviewee bluntly stated:

What happened in the appraisal itself … is, I'm being perfectly honest here: business as usual. We carried out our appraisal in exactly the same way as we carried out any other appraisal. No interaction with ministers or DH in terms of what we should/shouldn't be finding (Interview, NICE executive director, January 2012).

It may hence be suggested that although a ‘politicising’ issue network emerged, ministers did not seek to fully ‘politicise’—exert informal influence on—NICE's appraisal process. This evidence is limited in the sense that it does not account for informal phone calls or face-to-face meetings, but what evidence there is does not strongly support a counter-argument. One former NICE manager recalled informal teleconferences with DH officials about upcoming recommendations, but this was to manage media responses not discuss appraisal substance. A couple of interviewees also suggested DH may have contacted NICE by phone once specifically about Herceptin, but was flatly rebuffed. The question, then, is why was NICE apparently resilient to external pressures?

## Institutional Double Glazing

This section argues that ministerial intervention in NICE was avoided mainly due to institutional constraints reinforced by prevalent cultural norms—‘institutional double glazing’. This argument relates to the notion that ‘public organisations that are endowed with certain structural features … enjoy higher survival chances than those without these birth characteristics’ (Boin et al. 2010, 385). Lewis (2003, 143) argues that ‘political insulation’ or formal bureaucratic autonomy ‘decrease(s) the impact of changing administrations and changing majorities on the policies implemented by administrative agencies’. The notion of ‘insulation’ is particularly useful here, as it can be argued that the formal institutional design of NICE provided extra protection for scientific decision makers, in a form of ‘institutional double glazing’ that acted as an effective barrier to intervention.

Importantly, the appraisal of any health technology is not technically carried out by NICE but by the TAC *appointed by NICE*. Put simply, it can be argued that decision making is not ‘one-step’ removed from central government but *two steps removed*. DH appoints the chair of NICE, and meets senior NICE officials in high-level quarterly reviews, but NICE appoints the TAC, which *independently* assesses evidence, and senior NICE officials do not get involved with TAC decisions. As one interviewee bluntly put it, ‘if Andrew [Dillon, Chief Executive] went to the committees, they would all resign’ (Interview, NICE Non-executive director, January 2012). While it is important not to overstate the formal independence of TACs— NICE is legally responsible for final decisions—in terms of ‘*de facto*’ independence, TACs are highly autonomous, as one interviewee stated:

Once we have established a programme, it insulates the decision making from influences that are outside of the process to formulate the recommendations. There's no way DH or government can influence a decision that NICE are in the process of appraising (Interview, NICE Executive Director, January 2012).

One interviewee even suggested the whole point of NICE was to act as an insulator for the TAC:

We're not the ones who make the decisions, we're not in the room … On Herceptin, the decisions were made by a TAC sitting in a room, experts from throughout the health service. Obviously they're human beings who to some extent are influenced by what they see and read and [their] experience. But actually what we saw one of our jobs as being … was to … catch a lot of the rubbish and form a *protective barrier around the committee* so they could actually focus on the evidence and looking at the decision. In my time there I never went to a TAC meeting, it wasn't part of my role. My role was … to handle [the pressure] on their behalf and make sure that they could sit in *a kind of bubble* with all the evidence and look at it (Interview, former NICE communications manager, January 2012).

On top of this formal ‘barrier’, the appraisal process is highly formalised, and TAC procedures for evidence submission by stakeholders and methods of appraising evidence are specified in detail in several governance documents (NICE 2001; NICE 2004). The effect of this process was that once Herceptin had been referred, DH became just one of several ‘consultees’, and, as Table 1 shows, was contacted predominantly in that capacity once the process began. One interview in DH described their role as one of wearing different ‘hats’:

As well as the sponsor department, with a slightly different hat on we are a legitimate consultee in its work … once the topic has been referred we become a consultee in the development of that. Almost at the moment of that referral we swap hats … our primary focus was on making sure that NICE did as timely a job as possible doing what we needed to do as quickly as possible but in the appraisal itself it wasn't any different to what we would do for anything else (Interview, DH manager, January 2012).

This point suggests that DH's role was heavily circumscribed by official procedures, which required it to fulfil specific roles at different points. Although DH officials tried to rush the referral of Herceptin through as quickly as possible, any room for influencing NICE informally was squeezed out by rule-based procedures. As one interviewee put it:

The advisory bodies receive an evidence base from NICE and interpret it according to standard NICE guidelines … All of the comments that come in/responses are made public on the website. You can't do something and then hide it, without having to explain yourself to someone else (Interview, NICE Executive Director, January 2012).

Clearly, formal procedures are often followed because they coincide with informal norms or ideas about ‘good practice’ (Helmke and Levitsky 2004). Here, trust in clinical/medical expertise can be seen as an important underlying ideational factor reinforcing the more formal arrangements. The views of interviewees clearly portray a highly developed norm of deference and trust in NICE's clinical/scientific expertise, related to its long-serving senior management team and deference to medical science more generally. As one interviewee noted:

Two very strong features of NICE were credibility of the senior team and their stability over a long period of time, which is quite unusual … they secured the confidence of ministers and others, because in the end ministers appoint the chairman. There was a long period of stability with Sir Michael Rawlins and Sir Andrew Dillon and although there was always criticism (and this was the sort of thing that ministers would get involved in) … All those debates were going on but they were about the framework within which NICE operated rather than the decisions it was making … on the whole ministers left NICE to get on with it, and actually as its reputation built that became easier and easier (Interview, Former DH Senior Advisor, January 2012).

The elite interviews reflect a culture within Whitehall of deference to scientific/ medical expertise, particularly NICE's reputation as an international leader in reviewing evidence, as one interviewee put it: ‘The honest truth is what do ministers know about these things? It was an area of professional expertise’ (Interview, Former DH Senior Advisor, January 2012).

Beyond formal and informal institutions, there is also evidence that ministers and NICE officials enacted explicit strategies to deflect blame and prevent the crisis from escalating (Boin et al. 2009). For example, ministers blamed PCTs and clinicians for not supplying Herceptin sooner, as Hewitt (2005) argued that ‘As with other unlicensed drugs, it is down to individual clinicians to decide whether or not to fund Herceptin … PCTs must also be involved and will have to decide whether to support the clinicians' decisions and pay for Herceptin’. Similarly, Health Minister Jane Kennedy asserted that ‘it is for clinicians to decide, in discussions with patients, whether Herceptin is appropriate … the NHS … need(s) to make arrangements to provide Herceptin’ (HoC Deb 22 November 2005, 439:1359 and 1362). Moreover, as several interviewees noted, NICE was also developing its own ‘story’ about responding to the issue of ‘timeliness’, as illustrated by one Herceptin press release:

We are aware of the need for timely advice on the use of new medicines, particularly for life-threatening conditions such as cancer. The proposals we have set out mean NICE can deal with the current backlog much quicker than planned and that we will be able to issue guidance to the NHS rapidly in the future, once a drug is licensed (NICE 2005a, 2).

This ‘timeliness’ issue was identified by interviewees as an important theme in speeding up the introduction of the STA. These ‘narratives’ or ‘stories’ contributed to deflecting and diffusing the ‘blame risk’ associated with crisis situations (Hood 2011). In this case, however, they can principally be interpreted as methods for containing the crisis or stopping escalation to encompass wider issues of institutional legitimacy, rather than efforts to prevent intervention in this specific case.

## Conclusion

This article has made a distinctive contribution to analysing depoliticisation by examining how depoliticised bodies are *resilient* to external pressure for politicisation. In doing so, it has utilised cognate perspectives on policy networks and agencification to examine how NICE maintained its autonomy from DH during the Herceptin post-code lottery crisis. Specifically, it was argued that ‘institutional double glazing’: tight procedural rules and ‘double delegation’ critically reinforced by norms of deference to scientific/medical expertise within Whitehall mediated external pressures, such that politicisation was largely absent in this case. This article has important implications for further research on depoliticisation. Returning to the central ‘hooks’ of this article, it can be argued that it has implications in three areas: empirical, analytical and theoretical.

Firstly, the article has contributed empirically by examining the resilience of a depoliticised body (NICE) to external political pressure. This has implications for existing methodologies, in particular the most explicit one to date developed by Kettell (2008). Kettell (2008) develops a methodology which measures the success/ failure of depoliticisation based upon the societal reaction to certain depoliticisation tactics. Where society accepts a policy developed through depoliticisation, the tactic has been successful, whereas if society rejects the policy, depoliticisation has been unsuccessful. In a sense, the empirical analysis in this article looks at depoliticisation the opposite way round. Where an arm's length relationship is maintained despite external pressure, then depoliticisation has been ‘successful’, whereas if ALB autonomy is overridden, then the attempt to move towards an ‘indirect governing relationship’ has failed. Future research into the success/failure of depoliticisation might incorporate both analysis of ‘internal’ and ‘external’ success and failure, the effects of depoliticisation on society, and the effects of society on re-politicisation. Moreover, the identification of ‘institutional double glazing’ as an important factor in the resilience of depoliticisation also suggests further empirical research is needed into how politicisation is prevented. NICE, in this case, was a ‘successful’ form of depoliticisation in the sense that it was supported by a structure of formal institutional rules and informal norms that meant ministers did not seek to intervene in NICE's decision making processes. Future research may seek to further unpack the particular rules and institutional structures, their relationship with informal norms and ideas, and the extent to which these prevent re-politicisation, particularly in comparative analysis across policy sectors.

Secondly, this article has analytical implications in suggesting how the analysis of depoliticisation can be broadened, whilst retaining a core emphasis on the success and failure of depoliticisation tactics. It has suggested that engaging with cognate literatures may facilitate such an endeavour. This has implications for recent work, which has emphasised the importance of broadening analytical approaches to depoliticisation to involve a range of societal processes and mechanisms that support or reinforce institutional mechanisms deployed by governments, drawing on Hay's (2007) model (Wood and Flinders 2014). Empirical analysis has sought to develop heuristic indicators of Hay's forms of (de)politicisation through attempting to map the transition of issues onto and off of public and political agendas (Bates et al. 2014; Kuzemko 2014). One key argument of this article is that, in doing so, scholars are operating (implicitly or explicitly) within a tradition of research into state-society relations with a range of well-honed approaches that offer important insights. While deploying a broader framework of (de)politicisation is distinctive and intellectually stimulating, in order to avoid ‘reinventing the wheel’ scholars should consider the insights of cognate concepts, such as ‘security’ (Kuzemko 2014) and be cognisant of the potential for analytical overlap with well-established approaches. Further research might examine how other cognate literatures might be incorporated, and, moreover, how development of a fully-fledged analytical framework including a broader range of (de)politicisation processes may proceed in such a way that seeks to build upon, rather than displace, over half a century of research into state-society relations.

Lastly, and theoretically, this article has offered some more general insights into the depoliticisation thesis, in terms of how depoliticisation is effectively achieved. Returning to the assertion that delegation can be useful for politicians to institutionalise ideological preferences, the case demonstrates that this is far from straightforward. Rather it shows, as Rubin (2012) recently argued, ministers have to constantly re-assert the need for relying upon expertise, society does not merely accept it. Depoliticisation might hence be seen not as a one-way process of ideological assimilation, but as the institutionalisation of a particular mode of policy-making, emerging and being continually reproduced in a context of periodic contestation and struggle. Its success, ultimately, relies on whether the formal rules of delegated governance can survive political crises and media storms. Of course, given that only one case is examined here, the assertion cannot be made that depoliticisation works this way in all policy spheres. It can be noted however, that in very similar policy areas of ‘risk’, such as food safety, ministers very often face calls to intervene when a new crisis emerges, compared to areas like monetary policy, where intervention is rarely called for (even despite the 2007 financial crisis). Further comparative empirical study, as advocated by Hay (2014), may identify similarities and differences in how depoliticisation is effectively achieved more or less easily across areas of policy, and different political cultures.

## Notes

1. This includes senior ministers and civil servants at the Department of Health during this period (May 2005–August 2006), senior officials in NICE (Executive and Non-executive directors and members of the communications team), journalists who reported on Herceptin in national and local news, senior figures from Roche (the manufacturer of Herceptin), and legal representatives for Herceptin patients.

2. ‘Crisis’ is clearly a contested concept, but is defined in this article as ‘periods of political stress’ on governing institutions, most closely related to Alink et al.'s (2001, 290) concept of ‘institutional crisis’.
